# Simple lung retraction method for uniportal video-assisted thoracoscopic surgery

**DOI:** 10.1093/jscr/rjab465

**Published:** 2021-10-31

**Authors:** Yasushi Mizukami, Ryunosuke Maki, Hirofumi Adachi

**Affiliations:** Department of Thoracic Surgery, National Hospital Organization, Hokkaido Cancer Center, Sapporo-shi, Hokkaido, Japan; Department of Thoracic Surgery, National Hospital Organization, Hokkaido Cancer Center, Sapporo-shi, Hokkaido, Japan; Department of Thoracic Surgery, National Hospital Organization, Hokkaido Cancer Center, Sapporo-shi, Hokkaido, Japan

## Abstract

In conventional multiportal video-assisted thoracoscopic surgery, devices such as cotton-tipped applicators are used instead of graspers to avoid injuring the fragile lung tissue while stabilizing the lung and securing the surgical visual field. However, in uniportal video-assisted thoracoscopic surgery, which requires the simultaneous use of multiple instruments, the instruments tend to interfere with each other during the procedure because they share a single incisional port. Here, we describe a simple, easy and cost-effective lung retraction technique using cotton swabs to solve the problem. We present this technique and comment on its advantages, including decreased cost and improved surgical visualization.

## INTRODUCTION

Uniportal video-assisted thoracoscopic surgery (UVATS), which has been reported to reduce the duration of hospital stay and postoperative pain, is performed globally [1]. However, when performing lobectomy or segmentectomy using UVATS, we sometimes encounter problems such as the lungs encroaching on the surgical field, making adequate exposure of the vessels and bronchi difficult. Diego *et al.* reported the use of long suction cannula, vessel sealing devices and long graspers to provide an adequate field of view while performing the surgical resection [2]. However, grasping a fragile lung, such as an emphysematous lung, using a grasper can damage the lung tissue. Further, inadequate lung deflation leads to interference with the surgical field of view. When UVATS is performed, a single surgical assistant sometimes needs to hold two instruments in one hand, because the contralateral hand holds the scope. It is neither easy nor stable to hold two instruments in one hand. Here, we describe an alternative, simple, easy and cost-effective lung retraction technique using cotton swabs.

## CASE REPORT

**Figure 1:**
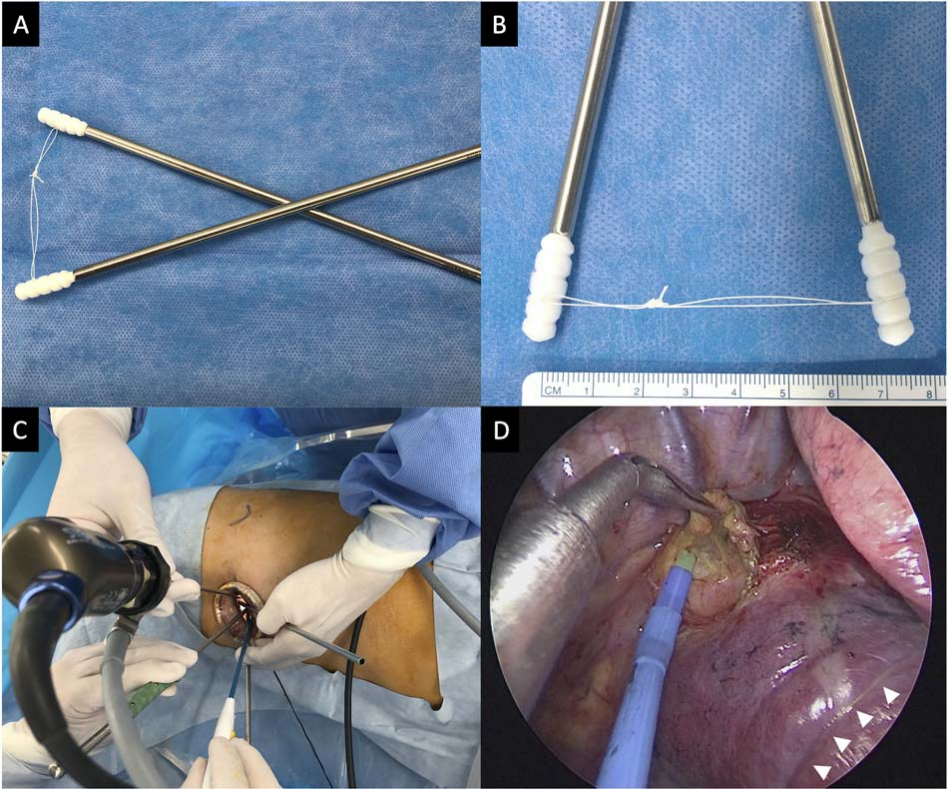
Connected cotton swabs and intraoperative view. (**A**) The connected cotton swabs. (**B**) The heads of two cotton swabs are connected by an ~7-cm long silk thread. (**C**) The forceps and the electrocautery are introduced from the incision by the surgeon. The assistant is holding the rigid 5-mm 30-degree video-thoracoscope and connected cotton swabs. The operator’s and assistant’s hands can be positioned with no interference between the instruments. (**D**) Intraoperative view of right subcarinal lymphadenectomy in the middle lobectomy. The cotton heads and silk thread is retracting the lung, creating a good surgical field (white arrowheads).

Ethics approval for this study was granted by the ethics committee of Hokkaido Cancer Center on 7 July 2021 (approval number: 03-08); the requirement to obtain informed consent directly was waived. Cotton swabs (Naruke Thoraco Cotton, Kenzmedico Co., Ltd., Saitama, Japan) are used as lung exclusion instruments in the field of general thoracic surgery. In our technique, the heads of two cotton swabs are connected by a silk thread about 7-cm long that the length can be adjusted as needed (Fig. 1). The sticks of the connected swabs are crossed and inserted into the thoracic cavity through the uniport. It is easy to move the connected swabs to retract the lung because their heads are not fixed. When the connected swabs are pulled apart adequately, the silk thread becomes taut, such that the cotton heads and silk thread can retract the lung, creating a good surgical field. Moreover, the assisting surgeon can easily and continuously stabilize the lung using one hand without the swabs slipping because of the connecting silk thread. In addition, the connected swabs are positioned away from the center of the surgical field, minimizing interference with instruments both inside and outside the thoracic cavity. The video shows lung stabilization using connected cotton swabs during left lower paratracheal lymphadenectomy (Supplementary Video 1).

## DISCUSSION

During UVATS, the energy device in one hand and suction device in the other hand are mainly used. Therefore, it is sometimes difficult to dissect the sheath of vessels using counter traction as in conventional multiportal VATS, because the suction cannula in the operator’s hand is used to maintain a good surgical field of view. In addition, achieving adequate lung retraction in the dissected area is sometimes more difficult during UVATS than during conventional multiportal VATS. Our connected swab technique does not interfere with either the surgeon’s visual field or the other instruments, while providing good lung retraction. We believe this technique is particularly effective for dissection of pulmonary vessels of the hilum, left lower paratracheal lymphadenectomy and bilateral subcarinal lymphadenectomy.

Moreover, since 2003, introduction of the Diagnosis Procedure Combination payment system in Japan has mandated a reduction in medical costs for each patient. Although cotton swabs are disposable, they are inexpensive and cost effective (~2 US dollars each excluding the metallic outer tube). We believe that our technique is effective for use during UVATS.

## AUTHORS’ CONTRIBUTIONS

Y.M. acquired the data and drafted the manuscript. R.M. and H.A. proofread the paper. All authors read and approved the final manuscript.

## CONFLICT OF INTEREST STATEMENT

The authors declare no conflicts of interest associated with this manuscript.

## FUNDING

No funding was received.

## ETHICS APPROVAL AND CONSENT TO PARTICIPATE

The author is accountable for all aspects of the work in ensuring that questions related to the accuracy or integrity of any part of the work are appropriately investigated and resolved. All procedures performed in studies involving human participants were in accordance with the ethical standards of the institutional and/or national research committee(s) and with the Helsinki Declaration (as revised in 2013). Written informed consent was obtained from the patient for publication of this manuscript. Ethics approval for this study was granted by the ethics committee of Hokkaido Cancer Center (approval number: 03-08).

## Supplementary Material

Video1_rjab465
